# Developing Industrial CPS: A Multi-Disciplinary Challenge

**DOI:** 10.3390/s21061991

**Published:** 2021-03-11

**Authors:** Martin W. Hoffmann, Somayeh Malakuti, Sten Grüner, Soeren Finster, Jörg Gebhardt, Ruomu Tan, Thorsten Schindler, Thomas Gamer

**Affiliations:** ABB AG, Corporate Research, 68526 Ladenburg, Germany

**Keywords:** Cyber–Physical System, Industrie 4.0, sensors, digital twin, artificial intelligence, industrial AI, switchgear, robotics, powertrain, process industry, operator support

## Abstract

Industrial Cyber–Physical System (CPS) is an emerging approach towards value creation in modern industrial production. The development and implementation of industrial CPS in real-life production are rewarding yet challenging. This paper aims to present a concept to develop, commercialize, operate, and maintain industrial CPS which can motivate the advance of the research and the industrial practice of industrial CPS in the future. We start with defining our understanding of an industrial CPS, specifying the components and key technological aspects of the industrial CPS, as well as explaining the alignment with existing work such as Industrie 4.0 concepts, followed by several use cases of industrial CPS in practice. The roles of each component and key technological aspect are described and the differences between traditional industrial systems and industrial CPS are elaborated. The multidisciplinary nature of industrial CPS leads to challenges when developing such systems, and we present a detailed description of several major sub-challenges that are key to the long-term sustainability of industrial CPS design. Since the research of industrial CPS is still emerging, we also discuss existing approaches and novel solutions to overcome these sub-challenges. These insights will help researchers and industrial practitioners to develop and commercialize industrial CPS.

## 1. Introduction

Sensors, actuators, and systems within industrial production facilities became more and more connected and were equipped with increasing computational power before Industrie 4.0 [1] was proposed. The requirement of a systematic approach for communication, data handling, cybersecurity, and distribution of software features grew. In industrial production today, the dominant approach to address this issue is Cyber–Physical Systems (CPS) [1,2]. CPS describes the connection between a physical asset, its digital twin and industrial applications, including the key technological aspects of communication and cybersecurity within the CPS. All the components and key technological aspects come together based on the purpose and use cases for which the CPS is designed.

Developing CPS for industrial applications, either designing new industrial CPS from scratch (“greenfield”) or upgrading existing physical systems (“brownfield”), is a challenging undertaking. The development is especially challenging because the consequences of a failing industrial CPS are typically severe, e.g., with respect to safety, productivity, cost, or company reputation.

**Figure 1:**
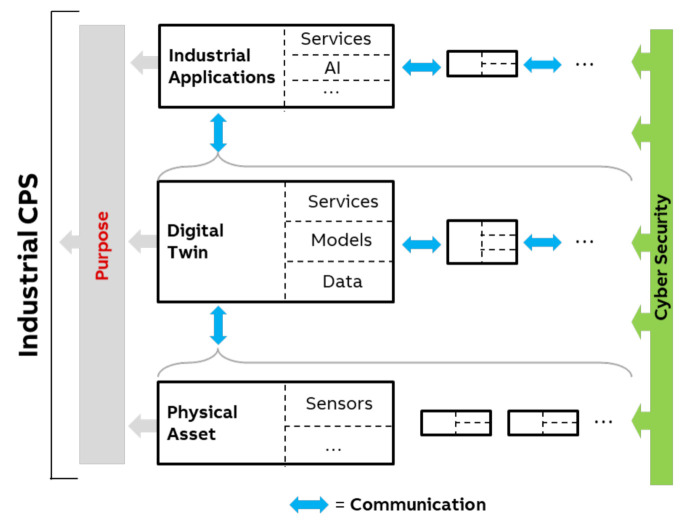
Concept of components and key technological aspects of a purpose-driven industrial Cyber–Physical System (CPS) and their relations to each other.

Figure 1 depicts a concept of how CPSs combine various components into one value-adding system. This concept is used throughout this article to structure the content and provide guidance of the challenges arising from developing CPS, and can be seen as a generalization of existing ideas and reference architectures, as we will explain in the next section in more detail. The CPS components have to communicate with each other whilst being real-time capable, highly available and reliable, as well as safe and secure. All these requirements—as well as the fact that such CPS always needs to be driven by a specific purpose—are strongly pronounced for industrial applications of CPS.

Traditionally, the components (physical assets, digital twins, and industrial applications) and other key technological aspects (communication and cyber security) of a CPS are developed independently of one another, as they have been utilized independently in industrial practice beforehand. Bringing diverse development paradigms, requirements, and approaches together is a major challenge for CPS providers in the industry, as the providers need to minimize R&D cost whilst providing a uniform experience to the user of the system.

This article aims at giving guidance for tackling the multi-disciplinary challenge of developing industrial CPS, by (i) describing CPS components and key technological aspects and their relations, (ii) providing insights into the R&D processes and recent technological advances of the components, (iii) breaking down the multi-disciplinary challenge into sub-challenges, and (iv) describing existing approaches and novel solutions to overcome these sub-challenges.

The presented findings in these four topics will help the scientific communities developing CPS, regarding both how to streamline their R&D processes, and how to achieve a faster deployment of their developments in large industrial systems. Our insights and recommendations are based on several years of industrial R&D for individual components of CPS as well as integrated CPS.

The remainder of this paper is structured as follows: we introduce CPS and specifications of industrial CPS in Section 2. In Section 3, we review four use cases of industrial CPS from different industrial domains. The roles of the three CPS components, namely physical asset, digital twin, and industrial applications, as well as the other key technological aspects of communication and cybersecurity in industrial CPS are reviewed in Section 4. In Section 5, we present the major sub-challenges in the development and operation of industrial CPS, plus existing approaches and novel solutions to overcome these challenges. Finally, we discuss the limitations of the paper and conclude in Section 6.

## 2. Specification of Industrial CPS

CPS are defined to be “systems of collaborating computational entities which are in intensive connection with the surrounding physical world and its on-going processes, providing and using, at the same time, data-accessing and data-processing services available on the Internet” [3]. While the areas of application of CPS range from medicine to smart buildings [4], we focus on industrial CPS applications which are in the scope of Industrie 4.0.

Figure 1 depicts a concept of how CPS combine various components into one value-adding system, based on Industrie 4.0 concepts. The concept in Figure 1 is a simplified view on CPS, focusing on the key components and technological aspects. As such, it is, to the best of our knowledge, a representative simplification—or generalization—of various existing definitions and reference architectures, such as RAMI 4.0 [5], the reference architecture model for Industrie 4.0, or SGAM [6], the Smart Grid reference architecture. Using the three-dimensional RAMI 4.0 as example, the concept in Figure 1 covers the “layers” dimension including six layers from asset to business while still abstracting from a clear mapping of components to layers. The same is true for the “hierarchy levels” dimension which is indicated by gray brackets abstracting from a clear mapping of components to hierarchy levels. The third dimension, “life cycle value stream”, is omitted in our concept in Figure 1 for simplification reasons. Still, we will address this aspect in the article when looking into the challenges and how to tackle those challenges (e.g., systematic management of Digital Twins in Section 5.1).

Figure 1 shows that a CPS is comprised of three key components: (i) physical objects (also denoted as physical asset), (ii) a digital twin of the physical object, and (iii) industrial applications, as well as key technological aspects: (iv) communication between all these components, and (v) cybersecurity within and across these components. For simplicity, only simple relations between these aspects are sketched to indicate that there are more complex relationships between these aspects in a CPS, both horizontally as well as vertically.

Horizontal communication can exist in all the key components. Between several physical assets within a CPS, such horizontal communication can be, e.g., mechanical or electrical connections. The layout of the connections between digital twins logically follows the layout of physical connections between physical assets. Furthermore, digital twins might include additional relationships, e.g., organizational structures or other logical relationships. Finally, industrial applications horizontally exchange data according to particular use cases.

Vertical communication within the CPS may include cross-component links between the industrial application and the physical asset, as well as additional information sources for digital twins, e.g., enterprise Information Technology (IT) systems or Engineering Technology (ET) data.

Clearly, there can be other options of relationships within a CPS, such as composite digital twins or deployment options to host a digital twin at the physical object or remotely. An additional aspect, which is not directly depicted in Figure 1, is the connection of multiple CPS into an overall industrial Cyber–Physical Systems of systems. We will address this aspect in Section 5.9.

For digital twins, myriads of definitions exist. For example, [1,7] defined the digital twin as a system containing both physical and virtual product. Ref. [8] described the digital twin as “a multi-physics, multiscale, probabilistic, ultra-fidelity simulation”. Digital twins are also considered to be a virtual representation of a physical asset. We build on the definition by Industrial Internet Consortium (IIC) and Platform Industrie 4.0 [9], which defines the digital twin as “digital representation, sufficient to meet the requirements of a set of use cases”, where the digital representation is defined as “information that represents attributes and behaviors of an entity” and an entity is defined as “an item that has recognizably distinct existence, such as a person, an organization, a device, a machine tool, a production line, a subsystem or a group of such items”.

In other words, a digital twin of an entity (a device, a subsystem, a plant) is the link between the cyber world and the physical world. A digital twin enables access to an entity’s life-cycle data, which can be Operational Technology (OT) data, ET and IT data. The data needs to be ingested and/or made accessible from relevant sources. For example, smart sensors can be attached to devices to solicit and provide OT data. A suitable connector needs to be established to existing databases to provide ET and IT data.

The digital twin offers Application Programming Interface (APIs) to industrial applications to access the data and hence offers new digital services [10]. Such services can be executed on a device, a local server (“edge computer”), or as a cloud computing service. The services focus on providing additional value to the industrial user and are developed in a user-centered approach [11]. Nowadays, these services utilize machine learning, optimization as well as domain-specific physical models to analyze the data.

The application of Artificial Intelligence (AI) is a promising example of industrial applications, which uses data from digital twins to learn from the history of entities and derive new conclusions and suggestions from the data. These conclusions and suggestions are useful in many ways, such as assisting the bidding and sales process or steering device performance by automatically adjusting set points.

In the physical part of a CPS, entities are connected to or interact with each other. A drive, a motor, a transformer and a pump, for instance, are connected to each other to form a drive powertrain (Section 3.4). The powertrain is also connected to other entities in the plant. Such connections and relations should also be reflected by the cyber part of the CPS, among the corresponding digital twins. The requirement of reflecting the connection between physical entities in digital twins leads to the formulation of a hierarchy of digital twins, from atomic ones to composed ones. It leads to horizontal interactions among the digital twins. Similar to physical devices, the corresponding digital twins may also be provided by various vendors, so interoperability across digital twins is necessary.

The components of the CPS are being held together by physical connectivity, which can be wired or wireless, ranging from Bluetooth to 5G [12], as well as information exchange, for example, via standardized information models [13] and technologies like OPC UA (Open Platform Communications Unified Architecture [14]). Furthermore, it needs to be clearly highlighted that physical sensors are important to collect data to be used in the CPS by digital twins and industrial applications.

Finally, security by design has to be an integral part of all considerations on CPS to achieve trust in the stability and functioning of the digital transformation and resulting digital solutions in the industry. With Industrie 4.0, for instance, flexible cross-company value creation networks will replace classical isolated production chains with largely hierarchical structure [15]. The evolution towards such cross-company networks will significantly broaden the attack surface, increasing the need for security solutions on all levels of a CPS. In Industrie 4.0, the need of having security solutions on all levels has been emphasized, e.g., by having security as a key aspect in the reference architecture model [16], acting “as a skeleton that carries and holds together all of the structural elements within RAMI 4.0”.

It is important to mention that security requirements and priorities in OT might be significantly different than the security requirements and priorities in IT, even though the basic technological building blocks might be rather similar. In OT, for instance, availability is the top priority protection objective, e.g., due to safety but also productivity and cost reasons. In IT security, typically confidentiality is the top priority protection objective, followed by integrity, and availability (CIA).

## 3. Examples for Industrial Use Cases

In the following, we present four examples of industrial CPS from different domains, to illustrate the typical status of CPS in today’s industrial practice along the proposed concept in Figure 1. The four examples were chosen to show the broad applicability of CPS in industry. Diverse as these examples are, they describe a typical benefit of CPS: New business opportunities often can be derived from CPS, such as for condition monitoring, system optimization capabilities, or simplified application development. In many cases, the technological basis for the new business opportunities includes innovative sensing, simulation, and increased system understanding. Use cases like these are important for the practical and conceptual development of CPS. Several statements in later sections have their origin in experiences from the four use cases mentioned in this section and similar real-world examples.

### 3.1. Medium-Voltage Switchgear

**Figure 2:**
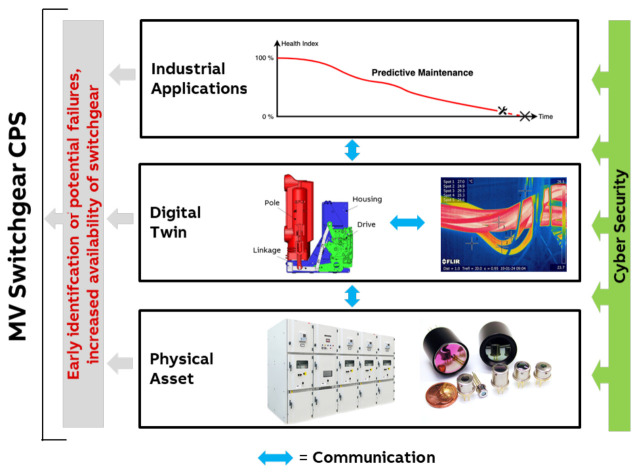
The CPS of a medium-voltage switch gear structured along the concept presented in Figure 1. The switch gear cabinets (bottom) are augmented with infrared sensor arrays. The live images, together with engineering know-how of the switchgear (e.g., CAD drawings of the mechanical parts), form a digital twin.

In a current research project, we developed a CPS for medium-voltage switchgear (Figure 2). The purpose of the CPS is continuous condition monitoring of the asset [17] to identify potential failures early and thus increase the availability of the switchgear. In order to do so, infrared camera sensors are built into the electrical switchgear. The thermographic images collected by the sensors are combined with first principle models of the mechanical and electrical behavior of the components of the switchgear to derive robust models for predicting required maintenance actions. For operators of switchgears, the robust models for predicting maintenance actions result in enhanced availability of the switchgear and strongly reduced maintenance cost. The digital twin thereby builds on general models and know-how from the engineering phase. This information is combined with the image data, which describe the online status, acquired by the infrared cameras.

The concept of a digital twin has to be flexible to represent individualized status data for various types of switchgear. It is planned that the digital twin should also include the functionality of predictive maintenance as a robust generalized model for all switchgear variants. In order to sense the usage, health status, and/or wear-out of specific switchgear over time, a robust image processing approach has to be developed [18]. Figure 2 summarizes the medium-voltage switchgear CPS. Details of the components and corresponding research challenges are discussed in [17].

The success of this type of CPS, from the point of view of switchgear and sensor suppliers, relies on the recent development of of suitable cost-effective infrared array sensors [19]. Furthermore, the relevant infrastructure and AI concepts, which can be used for image data transfer and processing, have been developed in the past few years in the context of Industrie 4.0 [20].

### 3.2. Industrial Robots

In a European research project (Productive 4.0), machine learning, optimization, CAD analysis, and experiments have been combined for making robot programming easier and faster, i.e., even more accessible to non-expert users. The result is an easy-to-use simulation environment for robot stations that takes into account the CAD data and the interactive inputs from the users in order to generate assembly task sequences automatically [21].

**Figure 3:**
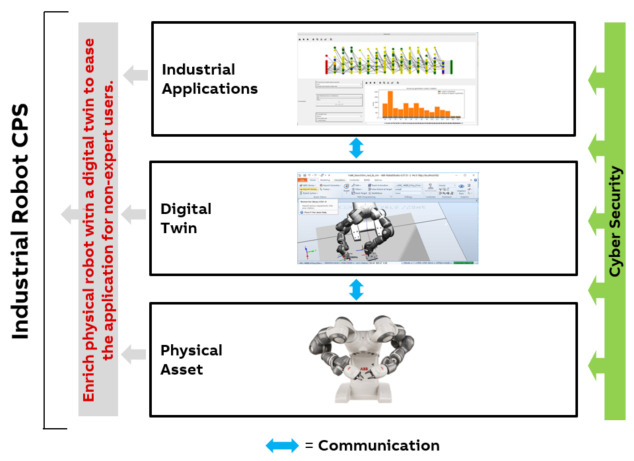
The CPS of an industrial robot structured along the concept presented in Figure 1. The purpose of the CPS for the industrial robot application [21] is to enrich the physical robot with distributed learning and optimization capabilities for non-expert users. Therefore, the digital twin consists of a CAD, simulation and a programming environment.

The simulation environment is extended to allow for easy understanding and adjustment taking constraints among product parts into account. Optimization techniques, in particular, a distributed learning approach, are utilized to achieve the flexible, robust, and reliable behavior of the robot [22]. For convenient deployment, automatic robot control code generation is also provided. Figure 3 provides an overview of the resulting CPS.

The challenge for the development of this industrial robot CPS is to achieve the consistent and efficient interplay of different interdisciplinary methodologies for non-expert usage. Therefore domain and expert know-how is the basis, combined with finding the best synchronization of harmonizing tools.

### 3.3. Process Plant Automation

**Figure 4:**
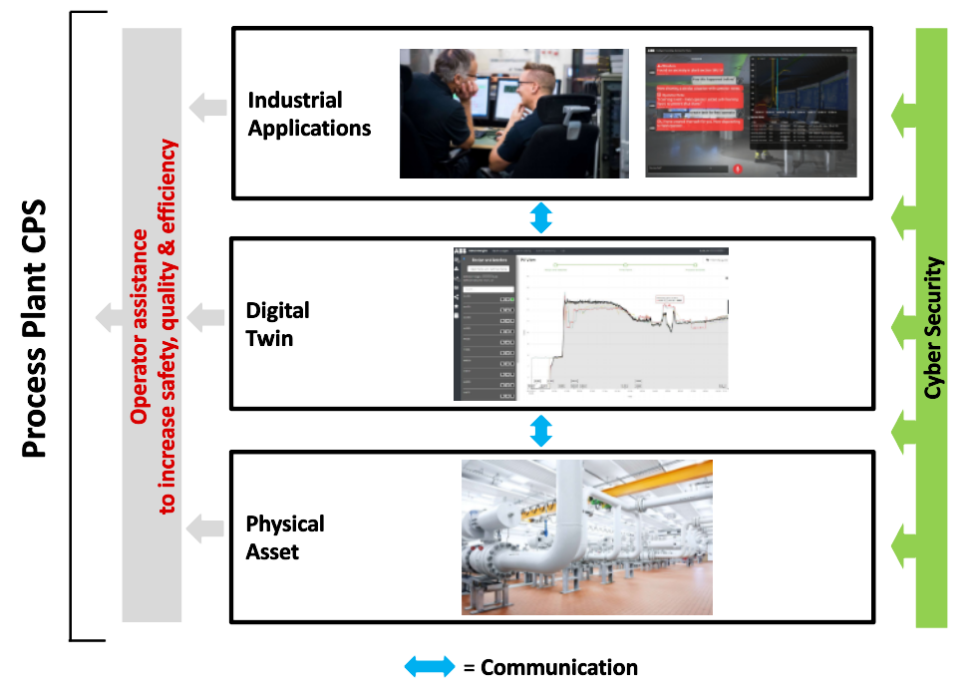
The CPS of a process plant structured along the concept presented in Figure 1. The purpose of the CPS for the process plant application [23] is to assist the operator by continuously providing essential information about the status of the process. The digital twin here is a tool to automatically identify deviations in the process based on process data.

Quality assurance of batch processes is complex and time-consuming. In particular, it is not easy even for experienced plant operators to keep track of the status of production in the process at any time, especially in critical situations. The purpose of the digital twin and industrial application components (Figure 4) is to assist the operator by continuously highlighting the essential information about the status of production.

The digital twin automatically identifies deviations in the process so that plant operators can correct them immediately. Numerous end users have been interviewed about their activities during batch analysis and their wishes for the visualization of the large amounts of data. The result is a sophisticated monitoring solution using machine learning and statistical methods tailored to the needs of process engineers and plant operators in batch production [23]. The solution is intuitive to use and enables users to identify process errors and their causes without requiring expertise in data analytics. The user interface can be easily integrated into process control systems, allowing plant operators to always have all essential information in view. The feedback from the usage of the tool in real plants, including experiments and real production operations, is used to further develop the digital services. The described system is thereby a step towards the introduction of virtual assistants in industrial production facilities [24]. These virtual assistants combine information from several monitoring systems, add additional analyses and predictions and make proactive services available to the user via multiple interfaces, e.g., natural language interface or integration into the control system [25]. The success of the Process Plant CPS depends first on the identification of the operator’s needs. Second, it is crucial for the development to combine black-box data analytics algorithms with tailored parameters and domain-specific know-how: this combination results in a sophisticated but successful approach.

### 3.4. Digital Powertrain

A powertrain is comprised of several assets: drives, electrical motors, shafts, bearings, gearboxes and loads, such as a pump or a fan. Powertrains are important assets in industrial systems such as process plants, mines or wind turbines. The digitalization of a powertrain leads to an industrial CPS with various physical and virtual components, which communicate horizontally and vertically w.r.t. the concept presented in Figure 1.

The mechanical parts of the CPS, including motors, gearboxes and bearings, have a considerable risk of failure whilst by default being an information black spot for traditional industrial sensing. Surveillance of mechanical parts in CPSs is a standard use case of sensorics for monitoring and optimization (M+O). As such, powertrain monitoring has been included in a recent NAMUR (User Association of Automation Technology in Process Industries) guideline [26], which in particular provides an extensive discussion of the business case of the corresponding CPS.

**Figure 5:**
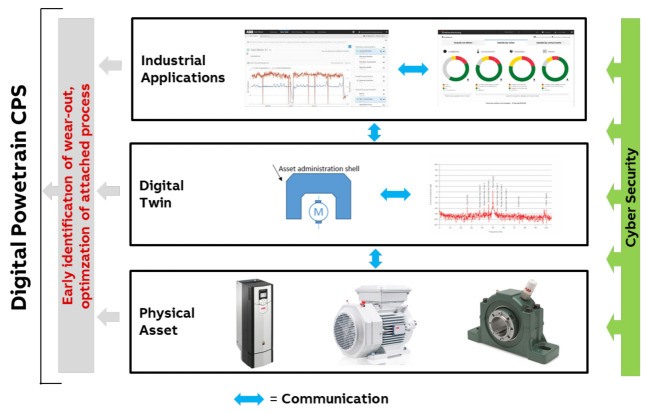
The CPS of a powertrain structured along the concept presented in Figure 1. External sensors are attached to a motor and bearing, e.g., using an AAS implementation to create a digital twin of their engineering and condition data, on which basis data-based services, like operations optimizations, are provided.

To overcome the problem that some mechanical parts are difficult to monitor, for example, an electric motor can be equipped with an autonomous sensor utilizing Bluetooth Low Energy (BLE) for communication and being battery-powered [27]. The sensor system will record and determine the condition of the motor and transmit the data via a cloud-based application on mobile phones. A similar sensor system can be attached to a bearing to record vibrations of the shaft in the bearing. The data is also stored and used by the same industrial application, which also accesses the data generated by the drive. The application can then provide insights into the overall behavior and condition of the powertrain and the production process associated with the powertrain. Figure 5 summarizes the CPS of a digital powertrain.

As described in detail in [26,28], the business case for this type of CPS puts strict constraints on the cost of sensing and communication infrastructure. As a consequence, specific sensor technology had to be developed or adapted, and communication protocols were taken from consumer goods markets, in order to benefit from economies of scale.

## 4. Key Aspects of Industrial CPS

Commonly, the various components of an industrial CPS are developed, commissioned and even used separately. The approach for developing each component is therefore different and the research strategies of the components are separated. Each component brings up its own challenges to the integration into a CPS. To elaborate on the current non-integrated situation, we discuss in this section the roles of the individual components in CPS.

### 4.1. The Role of Sensors in CPS

Obviously, digitalization creates a large demand for sensors. Sensors are inevitable in architectures of CPS, often in a pervasive way [29], since they make contact to the “physical” part of the CPS.

Nevertheless, taking process industries as an example, it is well-known that pervasive sensing cannot be accomplished with measurement instruments as they have been used before [30]. In today’s process plants, high-quality instrumentation is the basis of the automation system and sophisticated process control loops. Due to the high cost of the devices and their installation (from a few hundred up to several thousand dollars per measurement point), the number of such sensors needs to be minimized in a process. Maintenance and calibration structures of the sensors, as of today, are also not easily scaled up when the number of measurement points is planned to be increased significantly [31].

From the extensive list of new required sensor features as described in [30], we underline a few features, which are illustrated by the use cases in Section 3 and are relevant for the success of CPS.

(i) The sensors have to be low-cost (see Section 3.1 and Section 3.4 and Ref. [26]). Reduction of sensor costs can, for example, be achieved by taking off-the-shelf sensors from consumer goods, taking advantage of economies of scales, even when equipped with adequate packaging for industrial use cases. Other opportunities may include downscaling the resolution of sensors and the requirements of the level of accuracy.

(ii) Nevertheless, reliability and availability of the sensor systems often have to be enhanced significantly when the concepts of sensors from consumer goods markets are taken over to industrial use cases. Expected lifetime of sensor systems in industry is 5 years to 30 years while sensor systems for consumer goods have the lifetime of 2 years to 5 years.

(iii) The cost of communication infrastructure of sensor networks is often crucial for the overall economic success of a CPS concept. As an interesting development in the context of sensor development for industrial CPS, we mention open source hardware devices (e.g., Raspberry Pi or Arduino) which are now commonly used in R&D for easy CPS use case development. These devices take important roles as data sinks and edge computing devices. The concepts recently enter the industrial world in various forms [32,33], and the availability of affordable solutions increases.

(iv) Power supply cabling should be avoided if possible since the effort in industrial context may be large when the number of sensors grows. As a consequence, battery-powered or energy-harvesting devices with low-energy electronics will probably gain importance for the feasibility of a considerable number of CPS. Recently, reduced energy consumption becomes a key aspect for new sensor designs.

(v) Easy and flexible installation of the sensors or measurement instruments will be vital for widespread acceptance of CPS concepts in industrial practice [34]. Therefore, simplified mounting and non-invasive measurement concepts can be enablers for sensors.

(vi) It will be advantageous if the sensor concept allows a stepwise introduction of CPS in order to cultivate confidence in CPS. The stepwise introduction will vary in the size of the sensor network, the effort of planning and installation, the requirements regarding measurement quality, and the scale of technical and financial risk. Risk management is also supported by a reasonable communication concept, e.g., according to the idea of NAMUR Open Architecture (NOA) [35]. As outlined in [26], control of communication between Core Process Control (CPC) and local/cloud M+O systems (which are interesting candidates to be realized as CPS) is extremely important for industrial customers.

In summary, the first measurable benefit of the new type of sensors—and also of many types of CPS—will probably be seen in M+O. A possible next step, for instrumentation suppliers as well as for users, is to move a considerable number of CPS applications from traditional instrumentation to new sensor paradigms [30] under the framework of a CPS. The developments in sensor technology and industrial applications lay the foundation for the development in the level of maturity of Industrie 4.0 as they are discussed in Section 5.8. Pervasive sensing enhances the transparency of the production process which motivates developments of advanced communication concepts from both technical and economical perspectives.

### 4.2. The Role of Digital Twin in CPS

Although the notion of a digital twin is more associated with digitalization and the IoT era, digital twin existed before the IoT era under different names for different use cases [10]. For example, the concept of aspect object [36] specified digital twins for industrial systems, which contains information from various phases in the life-cycle of industrial systems. Naturally, early implementation of aspect objects did not take IoT-specific features and use cases into consideration.

The role of digital twins is becoming stronger in new industrial CPS systems because new categories of use cases emerge [10,37]. Several examples of these use cases are remote condition monitoring, cloud-based engineering, integrated tool chains, information exchange across value streams and beyond the boundaries of one organization. The use cases require systematic modeling and management of data at various levels of granularity.

In a CPS, OT data is gathered from sensors, IT and ET data are gathered from corresponding databases/systems and tools. The data need to be further cleansed and augmented with semantic descriptions. Correlations in the data need to be described in a machine-readable form to be used by (analytic) applications. The data also need to be complemented with models (built by simulation, machine learning or other modeling approaches) and suitable APIs need to be offered to the various applications to provide and process data.

All these aspects require a systematic definition and management of digital twins for a variety of use cases as well as data pipelines to work with them. Otherwise, companies may need to deal with excessive efforts of development and maintenance to deal with the consequences of ad-hoc solutions for data management.

Since industrial CPS can utilize applications and physical assets manufactured or even owned by different companies, a CPS can expand across the boundaries of one company. Therefore, the standardization of digital twins (e.g., data models, semantic dictionaries, and APIs) becomes mandatory to enable cross-company use cases. Current development in the standardization of digital twins is summarized as part of the sub-challenges in Section 5.

In summary, a digital twin is a key enabler for various Industrie 4.0 use cases because it facilitates better management of data and offers means to gain more insight on CPS and its constituents based on their life-cycle data. An effective design of digital twins serving a huge variety of use cases is nevertheless a challenge.

### 4.3. The Role of Industrial Applications in CPS

Data-enabled industrial applications exist in industrial practice for some decades. In the early years, these applications took the form of remote connections of industrial machines to the manufacturer. The data were usually analyzed semi-automatically and the owner and user of the machine were contacted if a problem, e.g., an undesired condition of the machine, arose. Nowadays, digital industrial applications include predictive maintenance, engineering automation and production optimization, as reviewed in cf. Section 3. The nature of the industrial applications, however, remained unchanged: industrial applications need to provide tangible values to the customer [38]. Furthermore, the need of engineers, service designers and software engineers collaborating in R&D processes of an industrial application persists and now data scientists also play a role.

**Figure 6:**
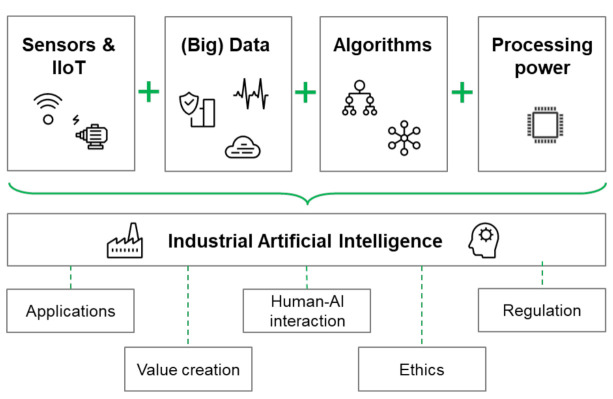
Pillars of industrial AI and adjacent topics of discussion [39,43].

The R&D of industrial applications was mainly driven by the better availability of more data, omnipresent computational resources, and improved connectivity. The current main technological driver behind industrial applications is industrial AI [11]. Figure 6 shows how industrial AI drives the R&D process of industrial applications.

AI technology is vastly advanced in the consumer sector, where we already see a multitude of AI applications, ranging from including image recognition and personalized advertisements to digital assistants. A direct transfer of these methods to industrial practice is not possible because an industrial environment puts different requirements on data-driven solutions [39]. However, albeit the first attempt of applying AI to industrial problems dating back to the mid-1990s [40], AI technology is now widely applied to industrial systems, including process automation, production optimization, and supply chain management [41,42].

Nevertheless, the view on AI technology, including its capabilities and scopes, remains unclear for most industrial companies particularly due to the diverse definitions of AI in academia. Here, we propose a simplistic definition of industrial AI in order to not overload application-driven discussions with technical details: industrial AI is the combination of the Industrial Internet of Things, available (big) data, AI-specific algorithms and sufficient computational power and infrastructure (Figure 6).

Current industrial applications utilizing AI focus on predictive maintenance [44] and decision support systems [45,46]. Furthermore, the application of AI technology to all layers of production systems [47,48] is being evaluated and AI-based virtual assistants are introduced [24,25]. A standard approach to the development of data-driven solutions is Cross-Industry Standard Process for Data Mining (CRISP-DM) [49] and its derivatives for industrial practice [11].

In the above-described use cases (Section 3), the examples of AI-based applications are the optimization-based assistance of robot programming, the prediction of required maintenance actions in switchgear or the optimization of the process operation by analyzing data from the powertrain CPS.

The data-driven solutions to industrial applications embedded in industrial CPS make use of the annotated data coming from the digital twins of multiple physical assets to provide insights into the overall condition of the production process and the associated physical assets. Hence, the solutions should be able to identify relations between data points from multiple digital twins, which may not be seen directly from the semantic enhancement of the data from a single digital twin.

In summary, current digital industrial solutions are mainly based on industrial AI and rely on useful data coming from the OT and IT systems. Novel sensors and digital twins can provide semantically enhanced and structured data for data-driven applications. The availability of enhanced and structured data enables more applications and increased reliability of algorithms. Therefore, it is reasonable to develop industrial applications integrated into CPS, not stand-alone.

### 4.4. The Role of Communication in CPS

As mentioned in Section 4.3, access to relevant data is a prerequisite to the application of industrial AI or industrial application in general. The digital powertrain use case in Section 3.4 gives an example of such a prerequisite. This prerequisite poses challenges to the R&D of industrial AI. In order to collect and assess such data from actual physical assets, effective and purpose-driven communication is a key technological aspect of a CPS.

On the one hand, industrial fieldbus technologies for communication are widely deployed and used for decades. These technologies can serve various application-specific needs such as reliability, robustness, and redundancy and might be a good basis for specific industrial use cases, such as process plant automation (Section 3.3). On the other hand, industrial communication is expensive because the multitude of various technologies causes huge efforts of engineering, integration, and management. The advance of industrial technologies [50] towards converged communication infrastructure has benefited greatly from evolving industrial-grade technologies, such as OPC UA, Time-Sensitive Networking (TSN, a set of IEEE standards to provide deterministic networking [51]), and Ethernet-Advanced Physical Layer (Ethernet-APL, intrinsic safe wiring and communication).

Meanwhile, consumer-goods-grade communication (e.g., BLE) increasingly influences R&D processes. For example, in the digital powertrain use case presented in Section 3.4, low-cost sensing and communication are required. A recent study of a NAMUR working group [26] on the performance of communication protocols in M+O sensor networks shows that communication is an important cost factor and hence decisive for the feasibility of many CPS (see also [28]). Additionally, 5G is another wireless technology that has arrived in the consumer market. This technology has great potential for industrial applications [52]. For example, ultra-reliable low latency or massive machine-type communication are part of the 5G roadmap. The possibility of local industrial 5G networks [53] makes this cellular technology more interesting for industrial use cases.

In summary, the importance of communication in CPS, both horizontally and vertically, can be seen from the use cases in Section 3. Still, there is no general answer to the most suitable, effective, and cost-efficient communication solution, as it depends on the purpose and constraints of a specific use case whether, e.g., established industrial fieldbus technologies, evolving industrial-grade technologies, or consumer-goods-grade technologies are the best fit.

### 4.5. The Role of Security in CPS

Security, of course, was always a quality attribute in the design of industrial solutions. Considering security requirements, industrial solutions have a different ranking of a priority than typical CIA (Confidentiality, Integrity, Availability) of IT systems. Availability, especially real-time capability, is ranked at the top due to safety, productivity, and cost reasons [54]. However, assumptions for cyber security in industrial systems were typically based on an “air gap”, where reliable, safe, and secure OT domain networks are assumed to be physically separated from other domains like office networks or the public Internet. Therefore, solutions are well established for the analysis of even very complex traditional systems with respect to its cyber security. For example, the analysis can be done based on threat modeling techniques [55] or a security development life-cycle process [56].

With a relatively small amount of inputs from domain experts, security engineers are able to understand the relevant inner workings of traditional industrial systems in order to carry out tasks for security analysis, such as threat modeling. For example, in a traditional system, it is relatively easy to reason about the role of a specific input. One can follow its usage and potential impact throughout the system in order to estimate the level of sensitivity of this input with respect to security and derive security requirements accordingly.

Modern CPS, on the other hand, are substantially different from these air-gapped systems. The reason is that CPSs assume converged networks, i.e., the communication infrastructure serves various applications and domains without any physical separation. A process plant automation system (Section 3.3), for instance, is typically based on such converged networks today. Similarly, usage of BLE communication and cloud-based applications in the powertrain use case (Section 3.4) increases the attack surface compared to traditional air-gapped systems. As a result of the complexity of modern CPS, cyber security analysis requires much more input from domain experts than what analysis of traditional systems may require.

Moreover, a key enabler for Industrie 4.0 is the availability of cross-company value creation networks [54]. These networks cover shop-floor, logistics, maintenance, and other aspects of the Industrie 4.0 life-cycle. As an example, the use case of process plant automation (Section 3.3) might comprise such cross-company value creation networks, with just-in-time production and logistics as integrated aspects. Industrie 4.0 life-cycle is highlighted by RAMI 4.0 and its three dimensions “layers”, “life-cycle value stream”, and “hierarchy levels”, and the security aspect applies to all three dimensions [16]. Both aspects of converged networks and cross-company networks broaden the attack surface, leading to an increased need for security solutions for all components of a CPS. Additionally, it requires security by design as an integral part of all phases to build trust in the stability and functioning of Industrie 4.0 and CPS systems.

Finally, security starts to play an increasingly important role in standardization over the last few years. Several examples are global ISA99/IEC 62443 on security for industrial automation and control systems [57], German VDI 2182 on IT security for industrial automation [58], Industrie 4.0 security guidelines by VDMA [59], and Baseline Security for IT systems and domain-specific security recommendations, both published and regularly updated by German Federal Office for Information Security. Additionally, we see new architectural concepts such as NOA [35].

In summary, security is becoming both more complex and more important with industrial CPS. As a consequence, efforts and challenges for achieving security increase and security is a non-negotiable integral part of the development of industrial CPS. Good news for CPS developers is that there are best practices available from IT security, and standardization is also starting to play an increasingly important role over the last few years.

## 5. Challenges and Solution Approaches

The development, sales, and operation of industrial CPS create several specific challenges, which need to be addressed in order to make CPS sustainable in a long term in industrial practice. For example, the challenges around future sensor developments for CPS arise naturally from the description given in Section 4.1 and, consequently, have been addressed there.

**Figure 7:**
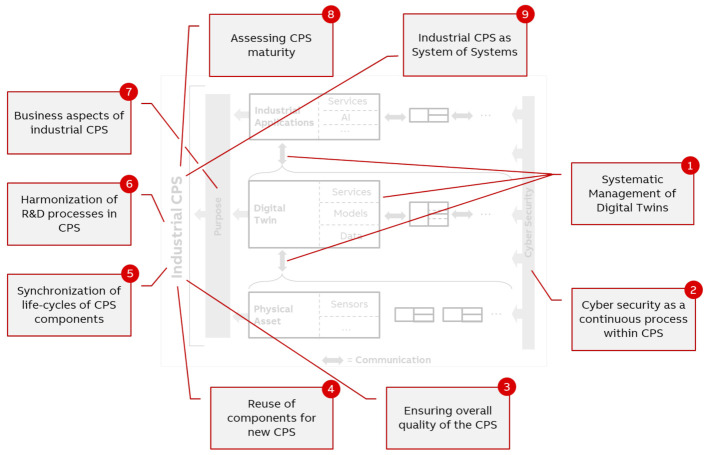
Identified major challenges in developing and managing industrial CPS, structured along the concept proposed in Figure 1.

In this section, nine major sub-challenges for developing and managing the CPS-structure above the physical layer are discussed. Figure 7 show the identified major sub-challenges and their relation to the concept of industrial CPS (Figure 1) and/or the entire CPS. This section also discusses solutions to address these challenges if applicable.

### 5.1. Systematic Management of Digital Twins

For the sub-challenge of systematic management of digital twins, we first describe the data silo problem in industrial systems, followed by a list of technical challenges that must be addressed in a common platform for digital twins. We then elaborate on the interoperability challenge across digital twins and a possible solution for it.

Different industrial devices have different phases in their life-cycles. Typical phases include type design, order/sales, engineering, production, installation, operation, and service. Life-cycle data of different devices are collected from different sources with different data formats and models which are considered suitable for the specific application.

There are several reasons behind the diversity of data sources. First, according to the “separation of concerns” principle, it is advisable to keep various types of data in different data storage systems due to the varying nature and formats of the data. Secondly, since different organizations may be responsible for different life-cycle phases of a device, naturally they may have their own data sources to manage the relevant data according to Convey’s Law [60]. Last, merger and acquisition activities cause different data sources, acquired from different companies.

The separation of data sources leads to the data silo problem. The lack of interoperability at multiple levels of data access (communication), data formats (syntax) and data models (structure and semantics) results in error-prone and time-consuming manual data exchange between the life-cycle phases, such as manual input of information or copy-pasting between different data models. The data silo problem also makes it difficult for analytics applications to combine data for harnessing.

Although a digital twin is claimed to address the data silo problem through accessing dispersed life-cycle data via unified APIs of the digital twin, the proper design of the digital twin, which enables it to effectively address the data silo problem, remains a challenge.

Here, we observe the following six challenges. (i) Communication and syntactic interoperability: to reduce maintenance costs and efforts of applications, unified APIs need to be designed for accessing data from diverse sources with various formats. (ii) Semantic interoperability: it is necessary to enable semantic alignment of various data sources so that applications can deal with heterogeneous semantics of data models from these sources. (iii) Data discovery: as the content of digital twins cannot be statically defined in advance, suitable mechanisms should be designed to dynamically detect new data sources and the associated data to make them available to applications. (iv) Data and security governance: offering integrated access to the otherwise dispersed data requires policies to govern data ownership, access rights as well as usage over the life-cycle of devices. (v) Similar to the challenge of syntactic and semantic interoperability, a conceptualization of mapping security-related aspects from the digital twin down to its data sources are required. For example, authentication and access-control information of an application toward the digital-twin can be translated into suitable technology-specific formats and passed over to the particular data source on the physical asset to enable an end-to-end security between the application’s users and data sources. (vi) Technology governance: different organizations may adopt different technologies to manage their data. With the advent of digital twins, we require a suitable platform to manage digital twins as well. In reality, there may exist multiple platforms, especially when different vendors offer their own platforms to manage their digital twins. Here, new organizational roles to manage platforms of digital twins and data pipelines will emerge.

In practice, industrial CPS are composed of physically interconnected components from various vendors within a production or a life-cycle context. Therefore, the corresponding digital twins need to exchange data in a way that reflects the physical interconnection of the components. To address the interoperability challenge of digital twins, Platform Industrie 4.0 in Germany has defined the specification of Asset Administration Shell (AAS) [61,62], which contains data models, APIs, and infrastructure components to manage asset administration shells.

Interoperability is “the ability to share information and services” [63]. Interoperability is characterized in multiple levels, such as technical or syntactic interoperability (data protocol, encoding) and semantic (data meaning within a given context) interoperability. Syntactic interoperability is addressed by a set of mappings of technology-independent AAS model to various communications and serialization protocols, such as OPC UA or RESTful HTTP APIs transferring JSON payload or XML files. The feature of interoperability allows AAS usage along the life-cycle of a CPS. For example, the file-based representation is used in the design phase and the API-based representation is used in the operation phase.

AAS provides methods for enhancing syntactic interoperability by annotations of exposed information with semantic identifiers. Those identifiers are based on standardized property catalogs (e.g., ECLASS (https://www.eclasscontent.com/ (accessed on 20 February 2020)) or IEC Common Data Dictionary (https://cdd.iec.ch/ (accessed on 20 February 2020))). Semantic identifiers allow identification of known properties, e.g., device weight or certain current temperature, in digital twins of previously unknown systems. However, the completeness of information for a specific use case, e.g., device monitoring, is not guaranteed. To deal with this problem, use case-specific sets of properties, data and object types for various use cases are developed in the scope of companion specifications for OPC UA and sub-model definitions for AAS, respectively. Examples include device identification and parameterization, models of the device status for monitoring purposes, as well as a readable technical specification.

Discovery of AAS and its contents (so-called sub-models which group atoms of information like variables, methods or collections) is addressed by a dedicated component, namely registries, in the infrastructure. Those registries are accessed using standardized APIs and allow listing and localization of technical AAS implementation and its sub-models via hyperlinks. Ongoing research and standardization activities also address further use cases like registries for searching or querying. Discovery of data sources, located between the physical asset and digital twin of CPS in Figure 7, involves data source-specific mechanisms, e.g., a Global Discovery Server (GDS) defined by OPC UA specification.

Looking into the specific challenge of mapping security-related aspects of a digital twin to a data source with specific technologies, Ref. [64] provides an example about how to integrate and implement AAS security concepts in OPC UA-based information models focusing on authentication and access-control information. Additional security governance challenges posed by data from digital twins will be further detailed in the next Section, in the broader context of cyber security being a continuous process within a CPS.

Regarding further governance issues, AAS can be adopted to exchange data with other companies in an inter-operable manner for brownfield scenarios [65,66], where companies already have their internal digital twins. As for greenfield scenarios, companies may start designing their digital twins based on the specification of AAS. Either way, several architectural and business questions related to data ingestion, as well as data, security and technology governance remain internal decisions of companies.

### 5.2. Cyber Security as a Continuous Process within CPS

For the sub-challenge of cyber security being a continuous process within CPS, we first give a rationale why cyber security is essential and needs to be a continuing effort from the beginning. Furthermore, we provide pointers to relevant guidelines, standards, and upcoming certifications, as well as motivate the need to constantly complement cyber security expertise with domain expertise.

One of the key challenges with cyber security of CPS is that it is impossible to achieve a secure system that remains secure. There are two reasons: first, there is no 100% security achievable [67]—security is a risk management process, which means it typically addresses security aspects most likely posing an issue in the context of the specific product or CPS. Therefore, security means both, to prevent security incidents by appropriate measures, as well as to detect and react to incidents [67]. Second, no digital system today remains unchanged regarding its interior (new features, upgrades, patches) as well as its exterior (system context, advances in attack technologies).

Therefore, cyber security is a continuing effort. In practice, however, R&D often applies a “feasibility first” approach, neglecting security in the first phase to create a working prototype quickly. This negligence should be avoided even in the early phases of CPS development, as it might require a complete—and potentially more costly—redesign when taking security into account in the later stage.

In CPS, cyber security is a continuing effort because the various entities, communications, and flexible system architectures in CPS require different mindsets across all dimensions of a CPS. The dimensions of a CPS are outlined for Industrie 4.0 in RAMI 4.0 [16] and the standardization also evolves; IEC 62443 [57] and VDI 2182 [58] are two examples. Furthermore, there are potentially upcoming regulations and certifications such as the EU-wide cybersecurity certification framework for digital ICT products, services and processes, under development by EU Agency for cybersecurity (ENISA) [68]. Specifically, it is inevitable that everyone being part of CPS development takes responsibility to consider—and basically understand—security during the entire process. The awareness of security by everyone in CPS development throughout the process also avoids the pitfall where one must start from scratch with new developments in regulations or certifications because basic security hygiene and, in the best case, security information management systems and secure development practices have been considered already.

With cyber security being a continuous effort during the complete life-cycle of a modern CPS, the challenge arises for the availability of expertise in cyber security and domain expertise during the complete life-cycle.

For traditional systems, cyber security is often performed by cyber security experts with the assistance from domain experts only at defined points in time during the life-cycle. Examples for this approach could be a threat modeling workshop during development or a penetration test prior to the release of the product. This approach not only results in time-constrained availability of security expertise, but also limits domain experts’ awareness of cyber security to these points in time.

As outlined in Section 4.5, the complexity of modern CPS requires cyber security expertise to be significantly complemented by domain expertise to achieve the necessary understanding of the CPS. With cyber security being a process, domain expertise needs to be constantly complemented by cyber security expertise. Therefore, the traditional approach of involving cyber security expertise when necessary is no longer suitable for modern CPS. Instead, a certain level of cyber security expertise needs to be part of the CPS development team at all times, either in combination with domain expertise or as a separate role in the team.

Due to the high and steady demand for cyber security expertise during the development and operation of modern CPS, the importance of cyber security and its role needs to be reflected and implemented on the governance level.

An example where these challenges can already be observed in more traditional systems is the introduction of AI and Machine Learning (ML). Even with domain expertise, it is a non-trivial task to analyze a system that uses AI or ML techniques in order to reason about the role and usage of a specific input in the final outcome. The result of the increased complexity in security reasoning is that domain experts need to be much more involved and trained in security analysis. Such a need not only drives up the cost of security analysis but also makes it less available due to budget constraints or simply the unavailability of the required expertise of domain experts or security experts.

### 5.3. Ensuring Overall Quality of CPS

For the sub-challenge of ensuring the overall quality of CPS, we first point to general requirements on industrial systems and how they affect CPS. Then, we provide recommendations to not only focus on functionality but also quality aspects during the development phase of CPS and lastly highlight a novel CPS-quality research aspect as an outlook for this sub-challenge.

Industrial systems underlie specific requirements regarding their quality, ranging from long-term robustness to strict safety and security guidelines [1]. Such requirements also carry over to digital services in industry [39]. The challenge for industrial CPS is that the other components may not fulfill these quality criteria when the requirements coming from one component needs to be carried over to other components of the CPS. For example, fast-evolving software components cannot achieve the long-term robustness of certified physical assets. Taking the digital powertrain (Section 3.4) as an example, a motor is expected to run without major problems for decades, whereas low-cost consumer sensing technology is designed to last for only a few years. Thus, consumer sensor platforms need to be adapted towards greater reliability and need to be easily replaceable because the lifespan of the sensor platforms will not match the lifespan of the motor. CPS, therefore, need to be managed such that the quality across governance domains, various vendors and evolving technology can be guaranteed.

It is therefore important to not only focus on the functionality of the CPS and its components during the design phase, but also to lay the foundations for the overall system quality including cyber security, maintainability, design-for-data, control of uncertainty in the physical layer, and scalability of calibration concepts. The quality focus may thereby interfere with agile development approaches (Section 5.6).

More recently, taking technical debts to speed up the time-to-market of software-enabled industrial systems comes to the attention of software engineers [69,70] and data scientists [71]. Taking technical debts means that developers take shortcuts during the development phase (taking a credit), which needs to be repaid with interest during later phases of development or product maintenance. It is important to balance between taking technical debts and fulfilling quality requirements of industrial systems as taking technical debt in one CPS component might jeopardize the overall quality of the CPS in the long run.

### 5.4. Reuse of Components for New CPS

This sub-challenge deals with the extension of the installed base to a new CPS fulfilling a new or advanced purpose without reinventing the wheel. We first provide the motivation for the challenge using the MV switchgear as an example, then identify major questions and related challenges. Novel solution approaches like iterative roll-out and simulation are outlined and refer to the use cases of process plant automation and industrial robot.

In the market sectors for automation and energy/mobility systems, sustainable solutions are required to be able to serve humans over a lifetime of several decades in the sense of circular economy. So far mainly dominated by conservative streams and nowadays affected by the energy and mobility turnaround, new solutions may integrate existing older (sub)systems or components, enrich them with new features and stay adaptable for updates for both greenfield and brownfield applications over the whole lifetime. Hence, the reuse of subsystems is an essential part of the user-and-purpose-centering development and affects the whole industrial CPS as shown in Figure 7.

The example of the MV switchgear presented in Section 3.1 is taken to highlight the questions regarding reuse and adaptation. Considering CAPEX and OPEX thoughts, an energy provider invests in switchgear with a lifetime of about 40 years. It has to be reliable and robust against disturbances and, in particular, it has to open and break electrical circuits, e.g., in case of faults that may not occur on a daily basis. The purpose of the switchgear is to ensure the safety of humans and other facilities. For reuse and adaptation, we should consider the following questions. (i) Changing requirements: can switchgear in the energy mobility turnaround still meet the requirement of resilience for fluctuating charging loads and dynamic wind energy? (ii) What are the minimal design adaptations needed to meet the requirements either by hardware or software updates and how can we easily certify that? (iii) What are further design updates required for digital services to avoid unplanned shutdowns or equipment failures and to enhance availability, reliability, safety, integrity and maintainability? (iv) Which hardware or software (sub)systems or components might be reused in a new CPS design?

For the extension of the existing system, extra sensors, data accessibility/interoperability /fusion from extra data sources are often dedicated to the respective mechanical or electrical components. These approaches rely on the appropriate design of the underlying algorithms, both data-driven and first-principle. ML methods, for example, suffer from an insufficient amount of failure samples in training data. This challenge of small data in particular for industrial applications is an active area of research. The challenges of maturity index and standardization are described in other sections.

Here we discuss a few solutions to the challenges of reuse. For instance, an iterative roll-out of digital services for industrial applications could be applied to cope with the challenge of small data. Exactly this approach is part of the process plant automation example in Section 3.3. Some implemented but not yet used features can be first validated with live data and value-adding later by a software update. Moreover, simulation can contribute to several aspects. It can be used to evaluate how the requirements are fulfilled and to determine what are the minimal changes needed to safely verify whether the feasibility and new constraints are met by the existing (sub)system but also by the overall new industrial CPS, cf. the industrial robot example in Section 3.2. The simulation could also be used to ease certification processes.

### 5.5. Synchronization of Life-Cycles of CPS Components

For the sub-challenge of synchronization of life-cycles, we explain how life-cycles of the components of CPS differ, highlight the user’s need to synchronize the operation phase and remind us to not forget the decommissioning phase of the life-cycle, as it is often omitted. We provide minor solution recommendations; however, the synchronization of life-cycles is a challenge that currently has not gained high interest from the research community.

The components of a CPS undergo asset life-cycles with different duration in the life-cycle phases. Besides the difference in duration within the R&D process, major differences arise during the operation, maintenance, replacement, and decommissioning phases. For example, first prototypes in software engineering are faster delivered comparing to mechanical prototypes. Digital twins, like a robot in simulation software (Section 3.2) may exist much earlier than the robot in the manufacturing environment (virtual commissioning). Mechanical components for industrial usage are usually built in the last several years or decades without significant degradation, whereas consumer-grade electronics components, e.g., in condition monitoring sensor systems (cf. smart sensor in digital powertrain, Section 3.4), may already degrade to fail after shorter periods of time especially when used in industrial production sites with tough environmental conditions (cf. switchgear CPS, Section 3.1). In contrast, cyber security components may never fail completely, but need to be regularly updated to ensure the protection. Similarly, ML models require periodic retraining to adjust to physical changes of the production equipment or the manufacturing process itself (cf. digital assistants in the process plant CPS, Section 3.3).

From a customer perspective, it is desirable to synchronize maintenance and upgrade activities to keep production downtime to a minimum and to schedule the synchronized activities along with the periodic maintenance of the rest of the industrial system.

When the decommissioning phase of an industrial CPS comes up, it will likely be worthwhile to archive data from the CPS for further usage by other systems and digital applications within the production facility o rat vendors’ side. In contrast to the pure recycling of mechanical and electric components, data archiving requires efforts from both the user and the application supplier. In terms of cyber security, it will be necessary to remove all data from electronics components, especially embedded systems. Overall, the life-cycle of the industrial CPS diverges into different decommissioning phases, with different actors and different duration.

To address these challenges, the phases of the life-cycle for each CPS component should be specified during the R&D process and the components should be designed in a way that especially the operation phase and the maintenance phase fit each other. Furthermore, the CPS should be adaptable throughout its life-cycle to changes in its environment [72].

### 5.6. Harmonization of R&D Processes in CPS

For the sub-challenge of harmonization of R&D processes, we first explain differences in R&D approaches for individual CPS components and review exemplary holistic R&D approaches. We then recommend designing a new light-weight interdisciplinary governing R&D process, which integrates the individual R&D processes of the CPS components, for CPS.

The research and development processes for the components of a CPS may vary substantially, ranging from waterfall-like approaches for the development of traditional industrial assets to agile and iterative R&D processes of AI-based services [11,49,73]. If aiming at developing an industrial CPS from scratch, these processes need to be harmonized by having clearly defined checkpoints and dependencies whilst keeping a maximum level of agility in the process to adapt to newly arising use cases and user groups.

Existing guidelines for the development of mechatronic systems were released in the early 2000s [74] and are thus not reflecting current technical developments such as digital twins, cybersecurity, and AI-based cloud services. More recent approaches propose integrated product development life-cycles [75]; but they are missing the specifications of the presented industrial CPS.

It, therefore, remains a major challenge to find a lightweight governing R&D process that harmonizes and integrates the established R&D processes of the various components. Thereby the different users of the CPS should be put in the center of the approach in order to adapt quickly to changing use cases, e.g., by providing mock-ups and prototypes as early as possible in the development process (test early, fail fast, succeed sooner). For example, when developing industrial virtual assistants (Section 3.3) the user of the existing OT and IT systems to which the virtual assistant attaches need to be integrated into the R&D process to ensure a smooth user experience, when using the systems together. Putting the user in the center of the R&D process will enable an agile working mode which benefits the early identification of failures and the design of a sustainable industrial system.

The development work of CPS is at the cross-section of computer science, physics, mathematics, and engineering (see e.g., the development of switchgear CPS in Section 3.1 where electrical engineers of the switchgear work side-by-side with physicists developing novel sensors and data scientist developing the predictive maintenance algorithms). For such an interdisciplinary development field, the formulation of targets is more challenging due to different expectations, development approaches, and vocabulary of the people involved. To address this challenge, new roles and responsibilities, e.g., data pipeline owner, API manager, interface program manager or system modeler, may need to be introduced. These roles will initially be ill-defined but will become more mature over time.

### 5.7. Business Aspects of Industrial CPS

For the sub-challenges related to business aspects of CPS, we first explain the need of a purpose of industrial CPS and recommend that every component of the CPS, in addition to the integrated CPS, should provide value to the user. Second, we suggest overcoming the challenge of integration cost when designing CPS by relying on open standards and a modular system design. The third challenge is that the business model for the integrated CPS needs to be synchronized when the components of the CPS have competing business models. Since the synchronization of business models is a topic of ongoing research, as an outlook, our recommendation is that data considerations should be put in the center of the business model innovation for CPS.

In the consumer market, a novel technology is unlikely to be adopted by the users just because it is fashionable [76]. In contrast, technical novelty in industrial assets, systems or services needs to come with an added value in order to be adopted by the market. As an example, cost-benefit calculations for real-world M+O-sensor network use cases are given in [26,28].

CPS, as still being a novel technology in the industrial space, therefore also need to have a purpose and deliver additional value in order to be sustainable in the market. Specifically, not only the overall CPS but also every component on its own needs to provide value. The reason is that not all components will be provided by a single vendor but may be sourced by an integrator from different vendors, whilst each vendor still keeps a direct technical and commercial connection to the end-user of the CPS. In the powertrain example (Section 5), the drive in the CPS fulfills a purpose on its own by efficiently powering the motor in the powertrain. The drive may thereby also send data to the control system of a process plant, which is not part of the powertrain CPS.

A technical yet business-related challenge for industrial CPS is the risk of high cost for integrating the components of the CPS because the user may fear being locked to a vendor long-term, if choosing a highly integrated CPS, potentially based on proprietary technology. Therefore, approaches like publication of interface descriptions, building on open standards like AAS [13,66] (as e.g., done also in the digital powertrain example, Section 5), and designing the CPS in a modular fashion, will increase the adoption rate and reduce R&D costs. Furthermore, opening for the integration of third-party components, even during the development process, will increase customer adoption.

Along with open standards, the usage of open-source hardware and software can be beneficial for reducing vendor lock-in risks and ensuring long-term maintainability of CPS hardware and software components. Open-source hardware is less common in industrial settings compared to a consumer environment due to specific application requirements [1,39], e.g., ingress protection, harsher environmental conditions, or strict safety regulation. Nevertheless, open-source hardware is reported to be used in industrial R&D for prototyping [77], and might increasingly be used, e.g., in industrial sensing in the future (cf. Section 4.1 and findings in [78]). On the other hand, the usage of open source software is more common for standardized industrial CPS building blocks ranging from operating systems to implementations of communication protocols, e.g., OPC UA [79,80] or AAS (https://www.eclipse.org/basyx/ (accessed on 20 February 2020)).

A further challenge lies in the harmonization of the underlying business model of each CPS component. As for product-like components, e.g., the physical asset or sensors, traditional industrial business models and business processes will be used. However, for digital twins or sensors integrated into digital services, service-like business models, which are novel to industry, are under evaluation. One example is the autonomous motor sensors used in the powertrain example (Section 5). Business models for digital services evolve from traditional services like maintenance contracts and consulting-like production optimization. Digital service business model development can quickly become very complex by itself [38].

Business models of complete industrial CPS are subject to research [17]. However, systematic approaches for developing industrial CPS-specific exist, e.g., by recombining existing business model patterns [81]. Business models for industrial CPS should focus on delivering the purpose and additional value to the end-users from the overall system. The delivery of purposes and additional values will likely include multiple roles, which utilize the CPS for different purposes, within a single customer organization.

In the future, industrial data and service ecosystems will influence the business success of industrial CPS. The design of the CPS, especially the digital services, should be cloud-provider agnostic in order to reach a broad range of customers. For the strategic and business positioning within a digital ecosystem, one could, for example, incorporate the method from [82] for positioning within industrial cloud platform ecosystems.

### 5.8. Assessing CPS Maturity

During the design phase of a CPS, its scope and requirements to CPS components have to be derived and fulfilled based on a certain use case (cf. Section 3) and the business purpose. One possible solution is to align the use case with an existing maturity index and to implement the recommendations according to the maturity index.

Another need for maturity assessment arises for existing CPS. As any other industrial systems, CPS will evolve over time—both caused by ongoing developments of new CPS as well as upgrades of installed CPS at the customer sites. It can therefore be of advantage to assess the maturity of a CPS, in order to identify components to improve. Such assessment is particularly relevant for industrial CPS, when compared to non-integrated industrial systems, as a certain level of maturity of the system is required to enable certain value-adding functionality.

In this section, we briefly review a state-of-the-art Industrie 4.0 maturity index and map the maturity stages to components of the introduced CPS architecture. While we picked this maturity index due to its domain fit, there are other maturity index concepts available. Technology Readiness Level (TRL), as initially defined by NASA [83], for instance, is wide-spread known in R&D context in general, and is seen as orthogonal to the Industrie 4.0 maturity index, i.e., every Industrie 4.0 maturity level might go from low to high TRL before moving to next maturity level.

Acatech defined six stages of maturity in their “Industrie 4.0 Maturity Index” in 2017 [84]. The first two stages, namely “computerization” and “connectivity”, for underlying physical entities are considered to be a basic condition for Industrie 4.0. Computerization means enabling the usage of (isolated) information technologies in the manufacturing process. The connectivity stage interconnects IT systems resulting in typically interconnected IT and OT layers within the company. Currently, most industrial systems are stuck at this level of maturity [84]. From the perspective of the introduced CPS concept (cf. Figure 1), both stages are hard requirements for data acquisition from the physical assets connected via an OT layer as well as for various IT and ET systems that are used as an information source for digital twins.

The third stage, “visibility”, aims to capture and access all the data around the physical asset and matches the objective of the digital twin components in Figure 1, such as breaking distributed information silos of various IT, ET, and OT systems.

Based on common information access, stages four, five and six can be tackled. The stages are called “transparency”, “predictive capacity” and “adaptability”. These stages fall into the components of industrial applications in our concept shown in Figure 1. Transparency aims at root-cause analysis of observed effects. Predictive capacity involves projecting observations into the future. Finally, the adaptability stage allows for automated decision-making based on observed and predicted effects.

Reaching higher levels of CPS maturity requires a complex interaction between multiple CPS which we discuss in the following section.

### 5.9. Industrial CPS as a System of Systems

Previous sections have covered the overview of the specifications of an industrial CPS (Section 2), the examples of industrial CPS (Section 3), and the description of the components (Section 4) of industrial CPS. An industrial CPS neither only consists of various components, nor only combines them loosely. From a systems engineering [85] point of view, each component is a system by itself as it provides value to customers on its own. For instance, an industrial CPS is a system of systems and overarching (Figure 7). This section outlines the respective sub-challenge and related thoughts on how to describe a CPS as a system of systems by combining various aspects of the article in a conceptual way. The description is a visionary contribution leading over to the last concluding Section 6.

The term “system of systems” (engineering) is not strictly defined [86,87]. The strength of an industrial CPS is that it can increase the value to customers by choosing the combination of single modular (sub)systems which benefit from each other to create a synergy that brings the overall system to the next level. Such a combination is always purpose-driven.

**Figure 8:**
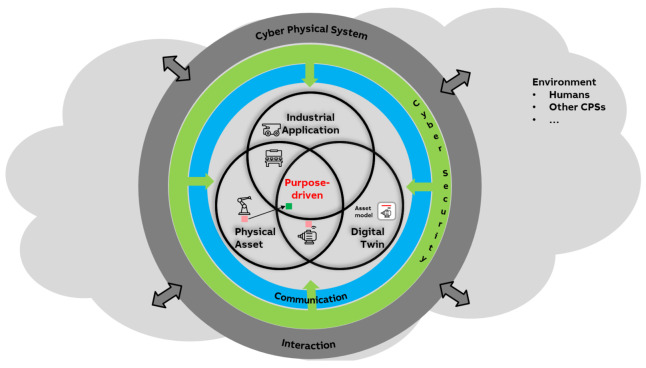
A CPS combines a vast amount of aspects and can be interpreted as a system of systems. Red marks: existing (sub)systems for existing purposes. Green mark: enhanced (sub)system for enriched purposes.

Figure 8 shows a possibly unconventional way to draw a facet of the industrial CPS compared to Figure 1. The idea is to place the purpose in the center of an industrial CPS. The purpose contains the value that an industrial CPS adds for humans, customers, and environment. A CPS fulfills its purpose with its simplest representation; it should not be more complicated.

However, industrial applications, physical assets, and digital twins can already consist of dedicated original tools to fulfill the new or enriched purpose. Figure 8 elaborates these points by comparing the two red-marked (sub)systems, namely a motor and a robot, as an example. Here, the robot is a pure physical asset and the motor in addition has an original digital twin. To meet the new purpose of the overall system, we need an industrial application and the adaptation of the (sub)systems. As presented in the example in Section 3.2, since we focus on the new value added by realizing the support of non-expert users, we can enrich the robot by adding new digital twin features. The additional digital twin feature shifts the red mark of the robot to the green mark in the center of Figure 8. On the other hand, we can use the motor without further adaptation to create purpose. Now we see how components and adaptations fit together in the overall system. This step may get complex and may require modularized setups, some of which can be reused in various CPS.

In Figure 8, the communication aspect wraps up the components and ensures a consistent glue between the (sub)systems. Obviously, the resulting structure logically contains the classic automation pyramid but also new concepts for the industrial IoT. For example, the cyber security aspect not only surrounds the industrial CPS but is inherently inside, intersecting with the communication aspect and interacting with the environment, which may consist of humans and other CPS. The related challenges, cf. Figure 7, concerning maturity level, modularity and standardization and further business aspects, the evolution over time for a circular and sustainable use, reuse of original (sub)systems, interoperability over different hierarchies, quality and technical debt are discussed in other subsections of this section. To summarize, an industrial CPS seen from a system of systems engineering perspective (i) brings interdisciplinary and even new knowledge, like quantum computing, and domain-specific approaches together, (ii) has to be reliable, resilient and efficient as a whole, (iii) is intuitive for user-experience while being complex, (iv) is conscious, i.e., even more than explainable, augmented and with on-time feedback embedded within the reality (v) takes into account sustainability concerning its effect on the environment and the (work of) humans.

When coming to potential solutions, co-simulation, e.g., simulation based on the functional mock-up interface standard [88], is a way to check the feasibility and the efficiency of the industrial CPS early in the development and to tackle the challenge of interdisciplinarity at the same time. By co-simulation, subject-matter experts can work efficiently using sophisticated approaches known in the respective field. To ensure intuitive usage, customers are involved in the development from the beginning. Augmented reality techniques can give the right insight to the expected behavior of an industrial CPS. A holistic approach can also give estimations on the effects of the industrial CPS and lead to novel and enhanced ideas.

**Figure 9:**
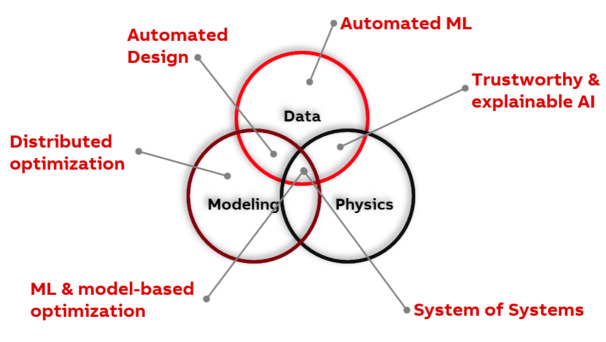
Physical asset and domain understanding, data and information description as well as first-principles or data-driven modeling have to be combined in a proper way to achieve the next level of sustainable and valuable industrial solutions, i.e., as simple as possible but not simpler.

Last, referring to Section 4.3 concerning industrial applications, we would like to give some extending thoughts. It is important to understand that humans behave consciously with all their collected experience and intelligence. For industrial CPS, we must distinguish between intelligence and consciousness. Intelligence in industrial CPS is now based on data (e.g., data models and ownership), sensors (e.g., sensor fusion), algorithms (e.g., machine learning, statistics, first principles), domain know-how, and processing power (e.g., edge, cloud, scalability, where the challenge arises). Consciousness, on the other hand, is not machine-intrinsic, yet important for cultivating users’ trust in the behavior of the industrial CPS. Thereby, an industrial CPS has to behave significantly better than a human because “to err is human” but errors are inexcusable for a machine. Therefore, we have to find synergies by choosing the right algorithmic treatment to achieve trustworthiness, maybe even consciousness of the industrial CPS as a system of systems (Figure 9).

To summarize, industrial CPS can be seen as a system of systems, where individual components collaborate and serve the purpose of the overall CPS. The purpose of CPS, such as consciousness and trustworthiness, is often beyond the functionality of a single component. Fulfilling such purposes requires the synergy of individual components.

## 6. Limitations and Conclusions

In this article, we introduced a concept of industrial Cyber–Physical Systemss and discussed the multidisciplinary challenges arising from such industrial systems.

The study presented in this article has the following limitations: (i) a multitude of definitions of CPS exists in scientific literature and industrial practice. We decided to go with a simplistic, yet practical, definition of CPS and its components to foster further industrial adaptation of CPS and illustrate the challenges in a transparent manner. (ii) The list of components of the CPS might not be complete. For example, safety of CPS was purposely neglected in this article because we acknowledge that safety requirements on CPS do not differ from other industrial systems and implementation will also not differ significantly from other traditional complex industrial systems. (iii) We reviewed the challenges for a majority of the components of the CPS. We did not discuss the challenge in communication separately in detail; instead, we addresses this challenge partially in the management of digital twins (Section 5.1). We also did not discuss challenges around industrial applications, but provided some insights in Section 4.3, where the industrial application is described as a component of CPS. (iv) Further challenges around the CPS as a whole and for all of its individual components exist but have not been addressed in this study as a result of prioritization because we aimed to provide an overview of the major CPS challenges. (v) We were not able to present solutions for all the challenges we reviewed as CPS is in an early technological phase. With the maturation of CPS in industrial practice, further solutions for the addressed major challenges will develop from future research.

Overall, a concept to develop, commercialize, operate, and maintain CPS in industrial practice was presented. Major challenges crucial to the long-term success of CPS in industry were identified and solutions, where available, were discussed. These insights may help researchers in the area of CPS to focus on the relevant topics and provide industrial practitioners insights for the strategy of development and commercialization of industrial CPS.

## Data Availability

Not applicable.

## Abbreviations

The following abbreviations are used in this manuscript:

AAS	Asset Adminstration Shell
AI	Artificial Intelligence
API	Application Programming Interface
BLE	Bluetooth Low Energy
CAD	Computer-Aided Design
CAPEX	capital expenditure
CIA	Confidentiality, Integrity, and Availability
CPC	Core Process Control
CPS	Cyber–Physical System
CRISP-DM	Cross-Industry Standard Process for Data Mining
ET	Engineering Technology
IIC	Industrial Internet Consortium
IoT	Internet of Things
IT	Information Technology
ML	Machine Learning
M+O	Monitoring and Optimization
NAMUR	User Association of Automation Technology in Process Industries
NOA	NAMUR Open Architecture
OPEX	operational expenditure
OT	Operational Technology
OPC UA	Open Platform Communications Unified Architecture
RAMI 4.0	Reference Architecture Model Industrie 4.0
R&D	Research and Development
TRL	Technology Readiness Level
